# Microwave ablation combined with apatinib and camrelizumab in patients with advanced hepatocellular carcinoma: A single-arm, preliminary study

**DOI:** 10.3389/fimmu.2022.1023983

**Published:** 2022-10-26

**Authors:** Xin Li, Qiao Zhang, Qiaorui Lu, Zhigang Cheng, Fangyi Liu, Zhiyu Han, Xiaoling Yu, Jie Yu, Ping Liang

**Affiliations:** ^1^ Department of Interventional Ultrasound, The Fifth Medical Center of Chinese People's Liberation Army (PLA) General Hospital, Beijing, China; ^2^ Department of Ultrasound, Maternal and Child Health Hospital of Guangxi Zhuang Autonomous Region, Nanning, China

**Keywords:** microwave ablation, apatinib, camrelizumab, combination therapeutic strategy, advanced hepatocellular carcinoma

## Abstract

**Purpose:**

The aim of this study was to assess the safety and efficacy of microwave ablation combined with apatinib [vascular endothelial growth factor receptor-2 (VEGFR-2) inhibitor] and camrelizumab [anti-programmed death-1 (PD-1) antibody] in patients with advanced hepatocellular carcinoma (HCC).

**Patients and methods:**

Patients (age, >18 years) with histologically confirmed HCC and refractory to at least the standard first-line therapy were enrolled from 2 September 2018 to 17 January 2022. They first received ultrasound-guided subtotal microwave ablation. Then, beginning at 7–14 days after ablation, they were given apatinib (250 mg once daily) and camrelizumab (200 mg once every 2 weeks) until unacceptable toxicity or disease progression or death. The coprimary end points were progression-free survival (PFS) and overall survival (OS).

**Results:**

Fourteen HCC patients with Barcelona Clinic of Liver Cancer (BCLC) B and C stages were retrospectively enrolled. At data cutoff, follow-up period ranged from 3.8 to 41.3 months (median, 17.4 months), and the median (95% confidence interval) duration of exposure (DE) was 6.4 (4.0–8.9) months. The PFS and OS were 10.8 (0–23.5) months and 19.3 (2.4–36.2) months, respectively. Three (21.4%) patients achieved a confirmed complete response (CR). Confirmed partial response (PR), stable disease (SD), and progression of disease (PD) were achieved in four (28.6%), four (28.6%), and three (21.4%) patients, respectively. The objective response rate (ORR) and disease control rate (DCR) were 50.0% (20.0%-80.0%) and 78.6% (54.0%-100%), respectively. The serious treatment-related adverse events included one (7.1%) case with reactive capillary hemangiomas (grade 4), one (7.1%) with hypertension (grade 3), two (14.3%) with elevated transaminase and bilirubin (grade 4), one (7.1%) with platelet count decrease (grade 4), one (7.1%) with hepatic failure (grade 4), and two (14.3%) with gastrointestinal bleeding (grades 3 and 4).

**Conclusions:**

Microwave ablation combined with apatinib and camrelizumab treatment in advanced HCC patients demonstrated intriguing clinical activity and resulted in durable antitumor responses and significantly improved PFS and OS. The combination therapy is well tolerated, enabling further clinical studies.

## Introduction

Primary liver cancer was the second leading cause of cancer-related deaths worldwide in 2020 (1). Hepatocellular carcinoma (HCC), the most common type of liver cancer accounting for 75%–85% of cases, is a highly fatal tumor, with a dismal 5-year overall survival (OS) of 18% (2). Around 70%–80% of HCC patients are initially diagnosed with HCC of an already advanced stage. According to the Barcelona Clinic of Liver Cancer (BCLC) staging system, which is widely used in clinical practice for developing therapeutic strategies (3), patients with more than three recurrence lesions and distant metastasis are classified as having reached an advanced HCC stage. Although early-stage HCC could be cured by liver transplantation, surgical resection, or ablation, the high recurrence and metastasis rates (nearly 70%) following such radical treatments adversely affect the long-term prognosis (4).

For advanced HCC, the first-line targeted treatment options currently available are sorafenib and lenvatinib, and the second-line targeted treatment options include regorafenib, bosutinib, and ramucirumab. The median OS for patients on targeted treatments ranged from 6.5 to 13.6 months, while the most objective response rates (ORRs) were less than 10% (5–7). Since 2018, immune checkpoint inhibitors (ICIs), namely, programmed death-1 (PD-1) and programmed death ligand-1 (PD-L1) inhibitors, have been available as a therapy option for advanced HCC with significantly improved prognosis, almost doubling the ORR (to the 15%–20% range) (8, 9). However, since HCC has the intrinsic dual features of an immune organ with innate immune tolerance and rich blood supply, the overall treatment efficiency has remained limited. More recently, combination therapy using antiangiogenesis and ICIs showed great superiority in the treatment of HCC. For example, the IMbrave150 study showed that, compared with the standard first-line target treatment with sorafenib alone, treatment with bevacizumab plus atezolizumab improves the progression-free survival (PFS) and OS in patients with unresectable HCC (10). Additionally, the study KEYNOTE-524 reported that patients with locally advanced unresectable HCC treated with pembrolizumab plus lenvatinib achieved an ORR rate of 46.0% (11). Also, a study of first-line treatments for unresectable HCC with hepatitis B virus (HBV) infection in Chinese HCC patients by the ORIENT-32 group reported that, compared with sorafenib alone, sintilimab plus a bevacizumab biosimilar could achieve significant improvement in OS and PFS (12). However, even with an ORR in the 25%–46% range for the novel combination therapies (11–13), there are still more than 50% of patients with advanced HCC who will not benefit from these combination therapies. Thus, finding novel therapy strategies for the efficient treatment of advanced HCC remains an important medical issue.

For early-stage HCC, a variety of local ablation techniques, including radiofrequency, microwave, laser, and cryoablation, are available as curative treatment options and are widely applied with comparable clinical efficiency. Microwave ablation (MWA) has several advantages over other ablation methods, including faster heating, higher thermal efficiency, and larger tissue necrosis. Thus, MWA is increasingly the favored technique to be used for radical treatment in the early stage and for palliative tumor reduction therapy in advanced HCC (14, 15). In previous studies, thermal ablation was reported not only to immediately extinguish necrotic tumor and reduce tumor load but also to regulate the local immune microenvironment *via* enhancing the expression of PD-1, activating cytotoxic T lymphocytes, and inducing antigen-presenting release (16–19). Interestingly, ablation itself was shown to induce a locally ablated zone with a peripheral immune response, especially 3–8 days after treatment (20). Additionally, Duffy et al. (21) reported that tremelimumab [a Cytotoxic T Lymphocyte-Associated Antigen-4 (CTLA-4) inhibitor] combined with radiofrequency ablation, a potential novel treatment for advanced HCC, led to an accumulation of intratumoral CD8+ T cells. Moreover, combined systemic plus locoregional therapies that we previously reviewed may have synergistic effects without overlapping toxicity that can improve prognosis in advanced HCC (22).

Given the above lines of evidence, we conducted a clinical trial of MWA combined with apatinib [a vascular endothelial growth factor receptor-2 (VEGFR-2) tyrosine kinase inhibitor (TKI)] and camrelizumab (an anti-PD-1 monoclonal antibody) for the treatment of advanced HCC patients and evaluated the safety and efficacy of the combination treatment.

## Patients and methods

### Patients

Twenty adult (aged ≥18 years) patients with liver malignancy lesions who received MWA combined with apatinib and camrelizumab treatment were retrospectively enrolled in this study from 2 September 2018 to 17 January 2022. We used 11 more eligibility criteria, including Eastern Cooperative Oncology Group performance status (ECOG PS) score of 0–1; HCC histologically confirmed and refractory to at least the standard first-line therapy; BCLC B and C stages; Child–Pugh A or B classification (score ≤7 points); life expectancy of at least 3 months; hepatic function ≤twice the upper limit of the normal value; creatinine ≤1.5 times the upper limit of the normal value; serum albumin level >25 g/L; platelet count >50 × 10^9^/mm^3^; prothrombin activity >50%; total leukocyte count >1.5 × 10^9^/mm^3^; and hemoglobin level >80 g/mm^3^. The four main exclusion criteria were a history of interstitial lung disease, pulmonary fibrosis, autoimmune disease, and liver transplantation.

The study was approved by the Ethics Committee of the Chinese PLA General Hospital (Beijing, China) and was conducted in accordance with the Declaration of Helsinki, Good Clinical Practice, and local laws and regulatory requirements. Written informed consent was received from all patients.

### Procedures

Twenty eligible patients received ultrasound-guided MWA, as previously described (14). The microwave system (KY-2000, Kangyou Medical, Nanjing, China) comprised an MW generator, flexible coaxial cable, and cooled-shaft antenna. A 2450-MHz or 915-MHz MW system was used with a typical power output of 50–60 W during MWA. For patients with advanced HCC (at BCLC B and C stages), who had larger and more lesions, even with venous thrombosis, we used an ablation planning system with three-dimensional visualization. With this system, we could display the location and spatial relationship of the tumor with the surrounding structures, quantify tumor size and volume, and predict the time-temperature profile of ablation, thereby improving its safety and effectiveness. This system also enabled the planning of the implantation route and accurate positioning of the ablation antenna. For a tumor adjacent to the vital organs (the intestine, gallbladder, biliary ducts), hydrodissection and thermal monitoring techniques were applied to protect the organs from thermal injury.

After ablation, the residual tumor was considered as the evaluated lesion. Then, apatinib (250 mg once daily) and camrelizumab (at the dose of 200 mg once every 2 weeks) were administered starting on post-ablation day 7–14 or when the laboratory tests meet the standard of systematic therapy after ablation. Interval imaging studies were performed every 8 weeks. Cases eligible for evaluation were defined as patients who had received at least two doses of camrelizumab and at least one post-baseline tumor response assessment using the modified Response Evaluation Criteria in Solid Tumors (mRECIST). The combination drug therapy was suspended or terminated upon detection of unacceptable levels of toxicity or disease progression. Apatinib or camrelizumab treatment was suspended following any adverse event (AE) of grade ≥3 until toxicity degree decreased to grade 1. If the required treatment delay was more than 4 weeks, apatinib was discontinued. Modification of camrelizumab dose was prohibited.

#### Assessment of treatment efficacy

Tumor response was assessed by investigators using mRECIST and reported along with the 95% confidence interval (CI). Tumors were assessed by contrast-enhanced computed tomography (CT) or magnetic resonance imaging (MRI) at baseline, then every 4 weeks until 12 weeks, and every 8 weeks thereafter. The coprimary end points were PFS and OS. PFS was the time from the first ablation included in the protocol until the first documented progression of disease (PD) or last follow-up. OS was defined as the time between the first ablation and the date of death or last follow-up. The secondary end points included disease control rate (DCR), ORR, and DE. DCR was defined as the percentage of patients whose best overall response was complete response (CR), partial response (PR), and stable disease (SD). ORR was defined as the percentage of patients whose best overall response was confirmed CR and PR. Duration of exposure (DE) was the time from the first ablation to the drug treatment termination or death or the end of follow-up.

#### Assessment of adverse events

Ablation-related AEs were assessed according to the Society of Interventional Radiology classification system for complications by outcome (23, 24). Drug-related AEs were reported according to the NCI Common Terminology Criteria for Adverse Events v4.0.

### Statistical analysis

The Prism software (GraphPad) or SAS Version 9.4 (SAS Institute, Cary, NC, USA) was used to perform statistical analyses (all descriptive statistics). We used the Clopper–Pearson method to calculate ORR and DCR with two-sided 95% confidence intervals. We used the Kaplan–Meier method to analyze PFS and OS. P < 0.05 was considered significant.

## Results

### Patient characteristics

**Figure 1:**
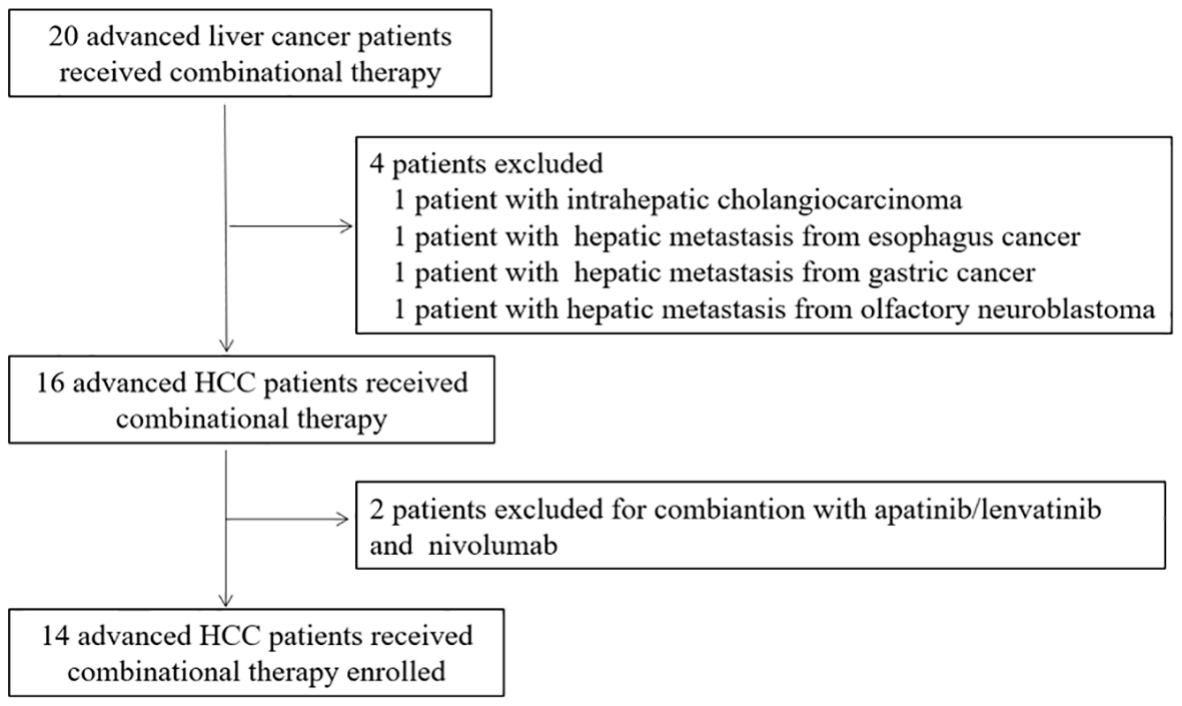
Trial profile for patient selection.

Twenty patients with liver lesions received the combination therapy. After evaluation, 14 HCC patients with advanced stage met all of the criteria and were selected (**Figure 1**). The baseline characteristics of the study population are summarized in **Table 1**. The median age of the patients was 59.5 years (range, 40–68 years); 12 patients were men. Eleven patients presented ECOG PS of 0. All 14 patients presented cirrhosis, 10 patients had hepatitis B, and all virus-positive patients were treated with antiviral medication at the time of enrollment. Eight patients were at Child–Pugh A (5–6 scores) classification, 12 patients presented with BCLC C stage, and 13 patients had elevated alpha-fetoprotein (AFP) levels (≥20 μg/ml). All patients had undergone ablation, 4 received surgery, 11 received transarterial chemoembolization (TACE) therapy, and seven had received prior sorafenib/lenvatinib therapy.

**Table 1:** Baseline characteristics of study population (n = 14).

Parameters	No. (%)
**Age, years, median (range)**	**59.5 (40-68)**
**Gender, n (%)**	
**Male**	**12 (85.7)**
**Female**	**2 (14.3)**
ECOG*	
**0**	**11 (78.6)**
**1**	**3 (21.4)**
**Liver cirrhosis, n (%)**	
**Yes**	**14 (100)**
**No**	**0**
**Child-Pugh score**	
**A (5-6)**	**12 (85.7)**
**B (7-9)**	**2 (14.3)**
**Etiology of HCC, n (%)**	
**Hepatitis B**	**10 (71.4)**
**Hepatitis C**	**1 (7.2)**
**No**	**3 (21.4)**
**Differentiated degree**	
**High**	**3 (21.4)**
**Median**	**7 (50.0)**
**Poor**	**2 (14.3)**
**No**	**2 (14.3)**
BCLC* stage	
**B**	**2 (14.3)**
**C**	**12 (85.7)**
**Prior therapies**	
**Surgery**	**4 (28.6)**
**Ablation**	**14 (100)**
TACE*	**11 (78.6)**
**Sorafenib/Lenvatinib**	**7 (50.0)**
APF* level (ug/ml)	
**< 20**	**1 (7.1)**
≥ **20**	**13 (92.9)**

*ECOG, Eastern Cooperative Oncology Group; HCC, hepatocellular carcinoma; BCLC, Barcelona Clinic Liver Cancer; TACE, transarterial chemoembolization; AFP, alpha fetoprotein.

### Efficacy

**Figure 2:**
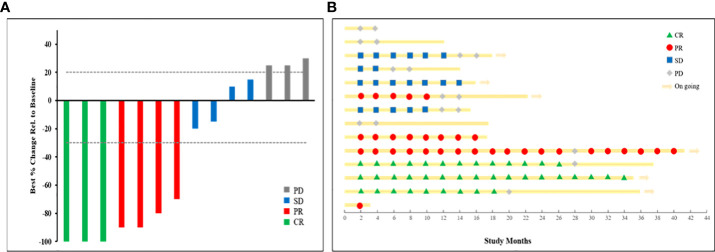
Treatment efficacy for the study population. **(A)** Waterfall plot of radiographic responses. **(B)** Swimmer plot of response status over study time.

**Figure 3:**
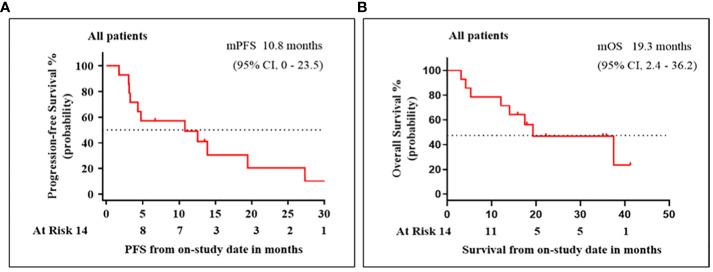
Patient survival for treatments with microwave ablation (MWA) in combination with apatinib and camrelizumab. Kaplan–Meier analysis of **(A)** progression-free survival (PFS) and **(B)** overall survival (OS).

**Figure 4:**
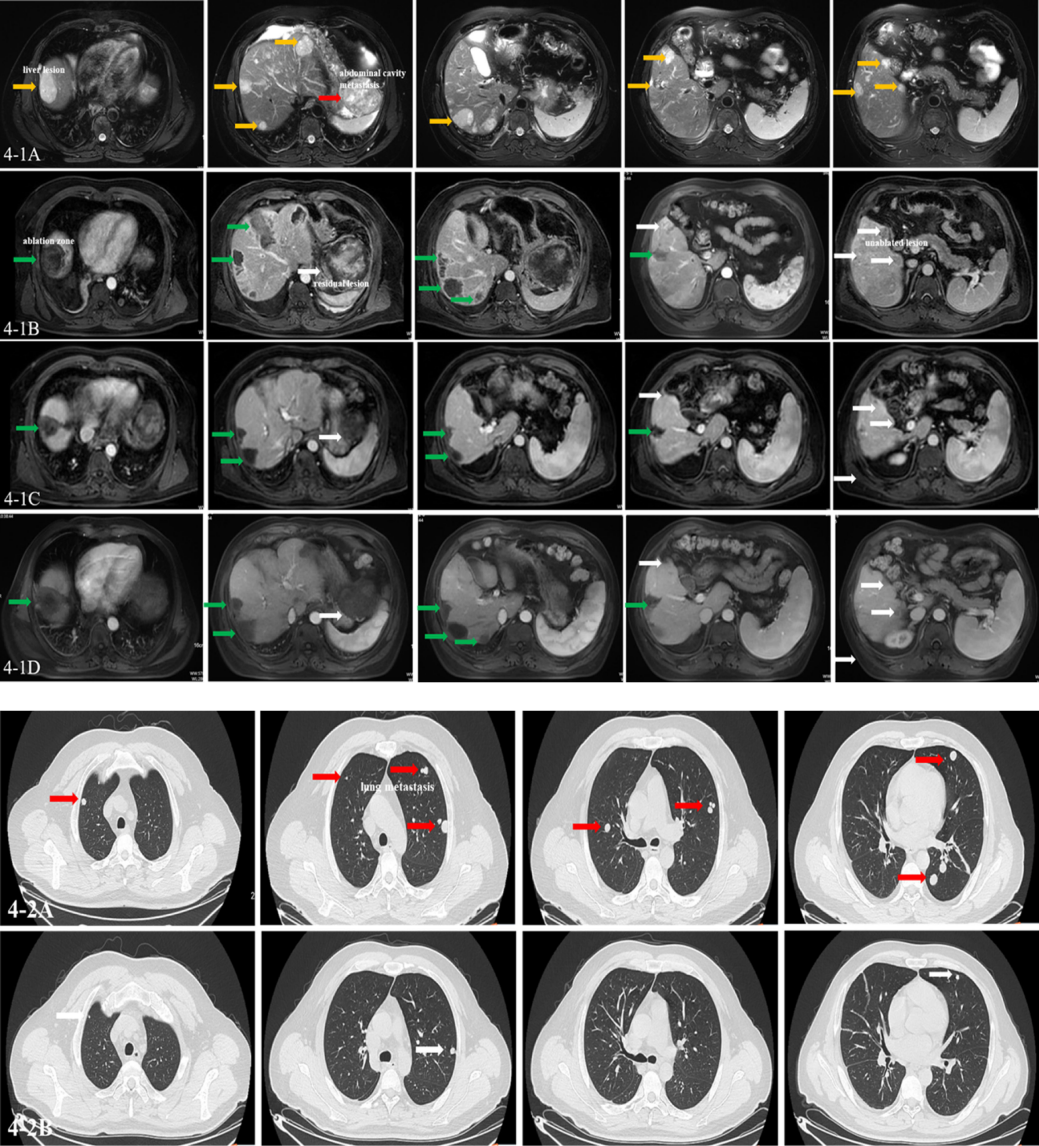
Imaging of clinical events. A man aged 45 years was diagnosed as having moderately differentiated hepatocellular carcinoma at Barcelona Clinic of Liver Cancer (BCLC) C stage, who received the combination therapy. **(4-1A)** The T2 phase of MRI images showing multiple lesions in the liver and abdominal cavity (yellow arrow: liver lesions; red arrow: abdominal cavity metastasis). **(4-1B)** The arterial phase of MRI images showing multiple ablated zone (green arrow), subtotal ablation of the lesions, and unablated lesion in the liver (white arrow) at baseline. **(4-1C, D)** The arterial phase of MRI images showing multiple ablated zone (green arrow) with no enhancement and the evaluated lesion shrunken with blood supply reduced (white arrow) after 6 and 12 cycles of combination therapy. The effect was evaluated as partial response (PR) by modified Response Evaluation Criteria in Solid Tumors (mRECIST). **(4-2A)** CT images showing multiple metastasis in the lung at baseline (red arrows). **(4-2B)** CT images showing lung metastasis shrunken obviously and some disappeared after six cycles of combination therapy (white arrow). The effect was evaluated as PR by mRECIST. Until now, the patient is alive with the imaging evaluated as stable disease (SD) lasting for 18 months.

By the end of the study follow-up period on 27 June 2022, the median (range) patient follow-up period was 17.4 (3.8–41.3) months and the median (95% CI) DE was 6.4 (4.0–8.9) months. Response to treatment data are summarized in **Table 2**. Three (21.4%) patients achieved a confirmed CR. Among the remaining 11 patients, PR, SD, and PD were confirmed in four (28.6%), four (28.6%), and three (21.4%), respectively. The median (95% CI) ORR and DCR were 50.0% (20.0%–80.0%) and 78.6% (54.0%–100%), respectively. **Figure 2** shows the efficacy data for the study population, including radiological responses (**Figure 2A**) and the quality and duration of objective responses (**Figure 2B**) during the treatment and follow-up periods. Seven patients died during the study follow-up period because of disease progression at 12.1, 14.0, 15.3, 17.3, 17.5, 23.2, and 37.5 months, and two patients died of AEs (hepatic failure and gastrointestinal bleeding) at 2.4 and 2.5 months. The median (95% CI) PFS and OS were 10.8 (0-23.5) months and 19.3 (2.4–36.2) months, respectively (**Figure 3**). The clinical efficacy of PR was achieved in a case of male aged 45-year-old who diagnosed moderately differentiated HCC at BCLC C stage and received the combinational therapy (**Figure 4**).

**Table 2:** Response to treatment (n/%).

Response	Investigator assessment (%)
CR*	**3 (21.4)**
PR*	**4 (28.6)**
SD*	**4 (28.6)**
PD*	**3 (21.4)**
ORR* (CR or PR)	**7 (50.0)**
DCR* (CR, PR, or SD)	**11 (78.6)**
Median PFS*, months (95% CI)*	**10.8 M (0.23.5)**
Median OS*, months (95% CI)	**19.3 M (2.4, 36.2)**
Median DE* (range)	**6.4 M(4.0, 8.9)**

*CR, complete response; PR, partial response; SD,stable disease; PD, Progressive disease; ORR, overall response rate; DCR, disease control rate; PFS, progression-free survival; OS, overall survival; DE, duration of exposure; CI, confidence interval.

### Safety

The ablation-related AEs included fever (>38°C), elevated white blood cell-to-neutrophil ratio, and elevated transaminase in all patients; elevated bilirubin in seven patients; and pleural effusion without catheter drainage in four patients. All of the AEs disappeared in 2–7 days with conservative treatment. Drugs-related AEs are summarized in **Table 3**. Six patients experienced at least one type of AE, and the majority were grade 2 (42.9%), requiring no medical intervention. The most commonly occurring drug-related AEs were diarrhea, reactive capillary hemangiomas (25), hypertension, decreased platelet and white blood cell counts, and elevated levels of aspartate aminotransferase (AST) and alanine transaminase (ALT). All grade 4 AEs, including decreased platelet counts, elevated AST/ALT, abnormal electrocardiography (ECG), immune pneumonia, immune hepatitis, and eventual hepatic failure, were developed by the same single patient. For three (21.4%) patients, treatment was terminated after grade 3 and 4 AEs (reactive capillary hemangiomas, hepatic failure, and gastrointestinal bleeding). One of the three patients was alive at the end of the study follow-up period; two patients died of serious AEs (hepatic failure and gastrointestinal bleeding).

**Table 3:** Treatment-related adverse events (n/%).

AEs* Among patients, N=10	Grade 2, No.	Grade 3, No.	Grade 4, No.
**Diarrhea**	**1 (7.1)**	**-**	**-**
**Reactive capillary hemangiomas**	**1 (7.1)**	**-**	**1 (7.1)**
**Hypertension**	**4 (28.6)**	**1 (7.1)**	**-**
**Platelet count decreased**	**3 (21.4)**	**2 (14.3)**	**1 (7.1)**
**White blood cell decreased**	**3(21.4)**	**-**	**-**
AST*/ALT *increased	**4 (28.6)**	**-**	**2 (14.3)**
**Total bilirubin increased**	**1 (7.1)**	**-**	**2 (14.3)**
Abnormal ECG*	**1 (7.1)**	**-**	**-**
**Hepatic failure**	**-**	**-**	**1 (7.1)**
**Gastrointestinal bleeding**	**-**	**1 (7.1)**	**1 (7.1)**

*AEs, adverse events; AST, aspartate aminotransferase; ALT, alanine transaminase; ECG, electrocardiography.

## Discussion

Currently, the efficacy of monotherapy (targeted therapy or ICI immunotherapy) for advanced HCC is limited, and US Food and Drug Administration (FDA)-approved first-line combination therapies are only available in a few regions of the world. Thus, there is an urgent need for alternative efficient therapies. Although existing combination therapies using chemical agents such as TKIs, as well as ICIs and antiangiogenic inhibitors, have been shown to be effective in treating advanced HCC (26), they are associated with the occurrence of intolerable AEs, low disease response rates, low DCRs, and relatively short OS. Thus, novel and optimized therapies are urgently needed (22).

This one-center, single-arm, preliminary trial was designed to explore the safety and efficacy of MWA combined with apatinib and camrelizumab in advanced HCC. To the best of our knowledge, this is the first report on a therapy strategy that combines ablation with TKIs and ICIs in advanced HCC patients. In this study, we combined localized MWA with sequential treatment with apatinib and camrelizumab commenced 7–14 days after ablation or when laboratory tests indicated readiness for systematic drug therapy. We showed that this treatment was efficacious and had a tolerable safety profile in patients with advanced HCC who had previously received multiple treatments. As a result of this combination therapy, tumor size shrunk and arterial blood supply to the tumor tissue was reduced in contrast-enhanced imaging evaluation (mRECIST). These were common phenomena and considered as criteria for treatment effectiveness. In contrast, conventional RECIST 1.1 criteria might underestimate treatment efficacy. For instance, in the same cohort of advanced HCC patients treated with a combination of avelumab and axitinib, the ORR was 13.6% according to RECIST 1.1 but 31.8% according to mRECIST criteria (27). In comparison, we observed an ORR of 50.0% (95% CI, 20.0%–80.0%) with the combination treatment in this trial, which was superior to the ORR reported for atezolizumab–bevacizumab (27.3%, 95% CI, 22.5%–32.5%) and sorafenib (11.9%, 95% CI, 7.4%–18.0%) (13). The comparison is also favorable with sintilimab plus a bevacizumab biosimilar combination used for treating Chinese unresectable HCC patients with HBV in the ORIENT-32 study, where the ORR was 25% (95% CI, 9.8%–46.7%) (12). Our result was comparable to that of the apatinib plus camrelizumab combination treatment (95% CI, 24.7%–75.4%), which is considered the best result to date (28). Intriguingly, there were three (21.4%) patients in our study who achieved a confirmed CR (lasting for 19.5, 27.3, and 35.1 months, respectively), which contrasts with no patient with a confirmed CR reported in the above study (28). The CR ratio in our study was also superior to the 5.5% CR observed in the atezolizumab–bevacizumab study (29). The CR ratio and the lasting time reported are the best to date. Overall, our results indicate that MWA combined with sequential drug treatment could achieve better efficacy than that with a therapy using a single drug or a combination of two drugs (28–31). As in a case report literature, in four HCC patients with advanced stage treated by TATI modality (Transarterial chemoembolization, ablation, tyrosine kinase inhibitors and immunotherapy), the longest survival time with 32 months and the shortest with 17 months were achieved (32).

The DCR found in this study (78.6%) was comparable to the 73.6% in the atezolizumab–bevacizumab study but superior to the 55.3% reported for sorafenib treatment. Among the DCR patients, more patients achieved SD and PR with the atezolizumab–bevacizumab treatment, while more patients achieved CR and PR in our study. Furthermore, the lasting time we observed with MWA combined with sequential drug treatment was longer than that with atezolizumab–bevacizumab and sorafenib (11, 13, 29). However, the DCR was 86% in a phase Ib study of unresectable HCC patients treated with lenvatinib plus pembrolizumab (11), superior to the DCR of our study. These results suggest that localized ablation combined with systematic therapy might have a synergistic effect in advanced HCC patients, but the mechanism of such an effect needs to be explored.

At the data cutoff date, where the median patient follow-up period (17.4 months) and DE (6.4 months) were relatively short, remarkably long median PFS (10.8 months) and OS (19.3 months) were achieved in this study. In comparison, the median PFS was 6.8 and 4.3 months in the combination therapy of atezolizumab–bevacizumab and sorafenib alone, respectively, in a phase 3 trial of atezolizumab plus bevacizumab in unresectable HCC (13). In another trial (GO30140), the median PFS was 5.6 and 3.4 months in the atezolizumab–bevacizumab and atezolizumab-alone groups, respectively (29). The lenvatinib plus pembrolizumab combination therapy (11) achieved a PFS (9.3 months) that was shorter and an OS (22 months) that was longer than those reported here. The ORIENT-32 study (12) reported results similar to ours. The median PFS, at 4.5 months, was much shorter for the monotherapy of apatinib in advanced HCC patients (30). These results indicate that the comprehensive combination therapeutic strategies will be prominent choices for advanced HCC.

Based on the above analysis, the clinical efficiency of localized MWA combined with sequential treatment with apatinib and camrelizumab may be the current best therapeutic strategy not only in short-term (PFS) and long-term (OS) clinical effects. The underlying mechanism might be as follows: 1) ablation could reduce the tumor burden greatly, as shown in this study (80%–90% of the whole tumor was destroyed by coagulation necrosis). Tumor burden is associated with the response to systematic therapy, especially with macrovascular invasion and metastasis (33, 34). 2) The local inflammatory response induced by thermal ablation will increase vascular permeability and possibly increase the efficacy of TKIs. 3) Ablation could enhance the local lesion and whole-body immunotherapy effect through enhancing the expression of PD-1 and activating cytotoxic T lymphocytes, which were considered key objectives for the combination of ablation and anti-PD-1 inhibitor. Other studies have shown that ablation and PD-1 inhibitor therapy significantly promoted the T-cell immune response, resulting in stronger antitumor immunity and prolonged survival in liver cancer (14, 35, 36). 4) The dual anti-PD-1/VEGFR-2 therapy has a durable vessel fortification effect in HCC and could overcome treatment resistance to either treatment alone and increase long-term prognosis in both anti-PD-1 therapy-resistant and anti-PD-1 therapy-responsive HCC models (37). The above findings provided important clues for our study design. Although a remarkable clinical efficiency was achieved in this study, the interactions of the three therapy components, ablation, apatinib, and camrelizumab, need to be further investigated.

Overall, apatinib and camrelizumab combined with MWA proved tolerable, with a manageable safety profile, in advanced-stage HCC. The incidence and severity of AEs observed with the treatment were consistent with the known safety profiles previously reported for the drugs (14, 28, 31, 38). Drug-induced grade 3 and 4 AEs were detected in three patients, and two recovered with conservative treatment with subsequent apatinib and camrelizumab treatment discontinued. Although adrenocortical hormone drugs were applied, one patient died of serious immune hepatitis and pneumonia. Therefore, close observation and follow-up as well as timely drug treatment should play a crucial role in combination therapy. Because of the good tolerance for AEs, the patients benefited from longer treatment times. This might explain part of the remarkable clinical efficacy of this combination therapy.

This study has limitations. First, the preliminary study is a single-arm study with a small patient sample, limited to a single institutional experience, and to a short follow-up time. Second, the intrinsic defects of a retrospective study are well recognized. Third, the potential selection bias cannot be excluded, as TKIs and immunotherapy are expensive and the patients with good financial status could afford even the three-treatment regimens domestically administered. In the meantime, all patients also received Chinese medicine (Huaier granule) or thymalfasin injection to improve their immune status (39). Furthermore, the expression of VEGFR-2, PD-1/PD-L1, and immune parameters should be analyzed, and the underlying mechanism for the interaction among the ablation, apatinib, and camrelizumab should be explored further. Finally, multicenter controlled studies with large samples and more predictive factors tested are needed to verify these striking preliminary results.

In summary, we combined MWA with apatinib and camrelizumab therapy in advanced HCC patients and demonstrated remarkable clinical efficacy, durable antitumor responses, and significantly improved PFS and OS. The good tolerance for these combination therapies enables further clinical studies. Then, more advanced HCC patients are expected to have the benefit of improved prognosis from the novel therapeutic strategy.

## Data availability statement

The raw data supporting the conclusions of this article will be made available by the authors, without undue reservation.

## Ethics statement

The study was done in accordance with the Declaration of Helsinki, Good Clinical Practice, and local laws and regulatory requirements. The study was reviewed and approved by the Ethics Committee of the Chinese PLA General Hospital (Beijing, China). The patients/participants provided their written informed consent to participate in this study.

## Author contributions

PL, JY, and XL designed the clinical study. XL, QZ, and QL collected and analyzed the data, XL wrote and submitted the manuscript. ZC, FL, ZH, XY, PL, JY, and XL performed the ablation. PL, JY, and XL performed the analysis, and PL and JY supervised the whole research process. All authors have read the final manuscript and declare no conflict of interest, and comply with the Journal Publishing Agreement.

## Conflict of Interest

The authors declare that the research was conducted in the absence of any commercial or financial relationships that could be construed as a potential conflict of interest.

The handling editor YX declared a shared affiliation with the authors, XL, ZC, FL, ZH, XY, JY, PL, at the time of the review.

## Publisher’s note

All claims expressed in this article are solely those of the authors and do not necessarily represent those of their affiliated organizations, or those of the publisher, the editors and the reviewers. Any product that may be evaluated in this article, or claim that may be made by its manufacturer, is not guaranteed or endorsed by the publisher.
